# CD52-Negative NK Cells Are Abundant in the Liver and Less Susceptible to Alemtuzumab Treatment

**DOI:** 10.1371/journal.pone.0161618

**Published:** 2016-08-25

**Authors:** Ryuichi Hotta, Masahiro Ohira, Toshiharu Matsuura, Izumi Muraoka, Panagiotis Tryphonopoulos, Ji Fan, Akin Tekin, Gennaro Selvaggi, David Levi, Phillip Ruiz, Camillo Ricordi, Rodrigo Vianna, Hideki Ohdan, Herman Waldmann, Andreas G. Tzakis, Seigo Nishida

**Affiliations:** 1 Division of Liver and Gastrointestinal Transplantation, Department of Surgery, University of Miami Miller School of Medicine, Miami, FL, United States of America; 2 Department of Pathology and Surgery, University of Miami Miller School of Medicine, Miami, FL, United States of America; 3 Cell Transplant Center, Diabetes Research Institute, University of Miami Miller School of Medicine, Miami, FL, United States of America; 4 Division of Frontier Medical Science, Programs for Biomedical Research, Graduate School of Biomedical Science, Department of Surgery, Hiroshima University, Hiroshima, Japan; 5 Sir William Dunn School of Pathology, Oxford University, Oxford, England, United Kingdom; 6 Cleveland Clinic Florida, Weston, FL, United States of America; Harvard Medical School/Beth Israel Deaconess Medical Center, UNITED STATES

## Abstract

**Background:**

T-cell depleting strategies have become an integral part of immunosuppressive regimens in organ transplantation. Alemtuzumab is a humanized monoclonal antibody against CD52, a cell-surface antigen on several immune cells. It has been suggested that lymphocyte depletion increases the risk of serious infections. However, this has not been observed with short-term alemtuzumab treatment in an organ transplant setting. For induction therapy using alemtuzumab following liver transplantation, we found that T- and B-cell numbers declined rapidly after alemtuzumab therapy; however, the natural killer (NK) cell number was sustained. NK cells are important effectors of innate immunity. Since the effects of alemtuzumab on NK cell functions, especially those of liver NK cells, are unknown, this study aimed to investigate this in detail.

**Methods:**

To assess the effect of alemtuzumab on NK cells, samples were obtained from 7 organ donors and examined by flow cytometry using Annexin V and propidium iodide. Phenotypical and functional differences within subsets of NK cells with different levels of CD52 expression were determined by flow cytometry and *in vitro* cytotoxicity assays.

**Results:**

CD52 expression on NK cells was lower than that on other lymphocyte subsets. The liver contained a large number of CD52^−^ NK cells compared with the peripheral blood. *In vitro* treatment of liver-derived NK cells with alemtuzumab did not result in cell death. In contrast, co-incubation with alemtuzumab induced cell death in peripheral blood mononuclear cells and non-NK cells in the liver. Furthermore, CD52^−^ liver NK cells were more cytotoxic and produced more IFN-γ than CD52^+^ NK cells after cytokine activation.

**Conclusion:**

The liver contains a large number of CD52^−^ NK cells. These cells are refractory to alemtuzumab and have robust activity. These findings indicate that CD52^−^ NK cells persist and could protect against infection after alemtuzumab-based lymphocyte depletion.

## Introduction

Alemtuzumab is a humanized, rat IgG1κ monoclonal antibody directed against the CD52 cell-surface antigen. CD52 is a glycoprotein expressed on approximately 95% of peripheral blood lymphocytes, natural killer (NK) cells, monocytes, macrophages, and thymocytes [1]. Lymphocyte depletion is expected to increase the risk of opportunistic infections [2, 3]. However, some studies have shown that the frequency of infectious diseases does not increase after organ transplantation [4–10].

For short-term induction therapy with alemtuzumab following liver transplantation, we found that T- and B-cell numbers declined rapidly after alemtuzumab therapy; however, the NK cell number was unchanged (S1 Fig). Similar results were previously reported for kidney transplantation [2, 3]. Therefore, we hypothesized that NK cells have an important role in resisting microbial attack during alemtuzumab induction for several months while T-cells repopulate. A clinical examination of some patients who underwent organ transplantation revealed that NK cells reconstitute the blood earlier than T- and B-cells after alemtuzumab treatment [5, 7]. The two mechanisms described above might offer a partial explanation of why lymphocyte depletion with alemtuzumab did not increase the incidence of serious viral infections following organ transplantation.

NK cells are important effectors of innate immunity and are identified phenotypically as CD3^−^CD56^+^ [4]. NK cells are functionally characterized by their ability to eliminate pathogen-infected cells and tumor cells without prior sensitization. This is achieved through the release of granules containing perforin and granzyme, and pro-inflammatory cytokines, especially interferon (IFN)-γ [5]. NK cells are abundant in the liver, in contrast to their relatively minor presence in peripheral lymphatics and other lymphatic organs in both rodents [6–9] and humans [10, 11]. In addition, liver NK cells differ from peripheral NK cells with regard to cytotoxicity toward cancer cells [10] and protection against viruses [12, 13]. In preliminary data, we fortuitously found that the liver contains a high amount of CD52^−^ NK cells, and we hypothesized that liver NK cells were refractory to alemtuzumab treatment because of their CD52 negativity.

In this study, we investigated the effect of alemtuzumab on the cell death of NK cells at a clinically relevant concentration, and characterized CD52^−^ NK cells by examining cytotoxicity toward tumor cells and IFN-γ production *in vitro* by using purified NK cells, especially from the liver.

## Materials and Methods

### Mononuclear cell preparation

The written informed consent for use of non-transplanted tissue for research and/or educational purposes from the donor or the next of kin was obtained. None of the transplant donors were from a vulnerable population and all donors or next of kin provided written informed consent that was freely given. The institutional review board of the University of Miami, School of Medicine approved this study. Peripheral blood (40 mL) and liver graft perfusate (2 L) samples were obtained from deceased liver transplant donors at the time of organ procurement. Liver mononuclear cells (LMNCs) were isolated from the liver graft perfusate, as previously described, because the proportions of LMNCs extracted from liver perfusates were almost identical to those collected using enzymatic dissociation [10, 11]. LMNCs and peripheral blood mononuclear cells (PBMCs) were isolated by gradient centrifugation with Ficoll-Hypaque (GE Healthcare, Pittsburgh, PA, USA).

### Flow cytometry

Flow cytometry (FCM) analyses were performed using an LSR II flow cytometer and BD ACCURI C6 (BD Biosciences, San Jose, CA, USA). The following monoclonal antibodies (mAbs) were used for surface staining of the lymphocytes: FITC-conjugated anti-CD3 (HIT3a; BD), anti-CD56 (B159; BD), anti-CD16 (3G8: BD), anti-CD94 (HP-3D9; BD), and anti-CD52 (HI186: BioLegend); PE-conjugated anti-CD52 (HI186: BioLegend), anti-NKp30 (P30-15; BioLegend), anti-NKp44 (P44-8.1; BD), anti-NKp46(BAB281; BECKMAN COULTER), anti-CD226 (11A8; BioLegend), anti-CD69 (FN50; BD), anti-TRAIL (RIK-2; BD), anti-CD107a (H4A3; BD), and anti-NKG2D (1D11; BioLegend); PerCP-conjugated anti-CD3 (SK7; BD), APC-conjugated anti-CD56 (B159; BD), APC-eFluor780-conjugated CD3 (UCHT1, eBioscience, San Diego, CA, USA), and eFluor 605NC-conjugated anti-CD16 (eBioCB16; eBioscience). Dead cells were excluded by light scattering and 4′6-diamidino-2-phenylindole (DAPI; Invitrogen, Carlsbad, CA) staining. Lymphocyte IFN-γ production was measured using a combination of cell surface and cytoplasmic mAb stains according to the manufacturer’s instructions. Briefly, 4 h after treatment with Leukocyte Activation Cocktail (BD GolgiPlug; BD), the cells were fixed and permeabilized with Cytofix/Cytoperm solution (BD) and washed with Perm/Wash Buffer (BD). Subsequently, aliquots were stained with mAbs against intracellular cytokines or anti-IFN-γ-PE (BD). FCM analyses were performed with the FlowJo software (Tree Star, Ashland, OR, USA).

### Detection of cell death after co-incubation with alemtuzumab

The viability of the residual cells was determined by FCM using the Annexin V-FITC Apoptosis Detection Kit (BD). The reagents were used according to the manufacturer’s protocol. LMNCs and PBMCs were exposed to various concentrations of alemtuzumab (0, 0.1, 1, 10, and 100 μg/mL) in RPMI 1640 medium without complement for different time periods (1 and 4 h). After the incubation period, cells were stained with 1 μL Annexin V solution and 5 μL propidium iodide solution for FCM analysis. Viable cells were negative for both Annexin V and propidium iodide. Dead cells were double positive for Annexin V and propidium iodide. We observed that Annexin V single positive cells were undergoing apoptosis.

### Isolation of NK cells

CD3^−^CD56^+^ NK cells were purified from LMNCs by magnetic cell sorting using the human NK cell isolation kit (Miltenyi Biotec, Bergisch Gladbach, Germany), according to the manufacturer’s instructions. The CD52 subsets of NK cells were sorted from purified NK cells with a BD FACS-Aria cell sorter (BD) using anti-CD3 (HIT3a; BD), anti-CD56 (B159; BD), and anti-CD52 (YTH34.5; AbD Serotec). Post-sorting purities were consistently >97%. NK subsets were then used for cytotoxicity and IFN-γ production assays. NK cell subsets were cultured in Dulbecco’s Modified Eagle Medium (Invitrogen) supplemented with 25 mmol/L HEPES buffer (Invitrogen), 10% heat-inactivated fetal bovine serum (Mediatech, Manassas, VA, USA), 100 U/mL penicillin, and 100 μg/mL streptomycin (Thermo Scientific, Waltham, MA, USA) (complete medium) with 1000 U/mL interleukin (IL)-2 (Proleukin, Novartis, Emeryville, CA, USA) for 2 days.

### NK cytotoxicity

The cell cytotoxicity assay was performed by FCM, as previously described [11]. Briefly, the target cells were labeled with 0.1 μM Vybrant^®^ CFDA SE Cell Tracer (Life Technologies, Carlsbad, CA, USA) for 5 min at 37°C in 5% CO_2_. The labeled cells were washed twice in PBS, resuspended in complete medium, and counted using trypan blue staining. The effector cells were coincubated at various ratios with target cells for 1 h at 37°C in 5% CO_2_. As a control, either target cells or effector cells were incubated alone in complete medium to measure spontaneous cell death. DAPI was added to every tube. Data were analyzed using FlowJo software (Tree Star). Cytotoxicity was calculated as a percentage using the following formula: % cytotoxicity = [(% experimental DAPI^+^ dead targets) − (% spontaneous DAPI^+^ dead targets)]/[(100 − (% spontaneous DAPI^+^ dead targets)] × 100.

### ELISA

IFN-γ production of NK cell subsets during culture was measured by ELISA (BioLegend). The supernatants were collected after incubation and stored at −80°C until further use. The IFN-γ ELISA was performed according to the manufacturer’s instructions.

### Statistical analysis

Data were presented as mean ± SD. The statistical differences of the results were analyzed by 2-tailed Student’s *t*-test (2 groups) or by analysis of variance (ANOVA) followed by a Tukey post-test (more than 2 groups), using the SPSS Statistics Version 22 for Windows (IBM Corp., Armonk, NY, USA). P values of 0.05 or less were considered statistically significant.

## Results

### The liver contained a large number of CD52^−^ NK cells

**Fig 1:**
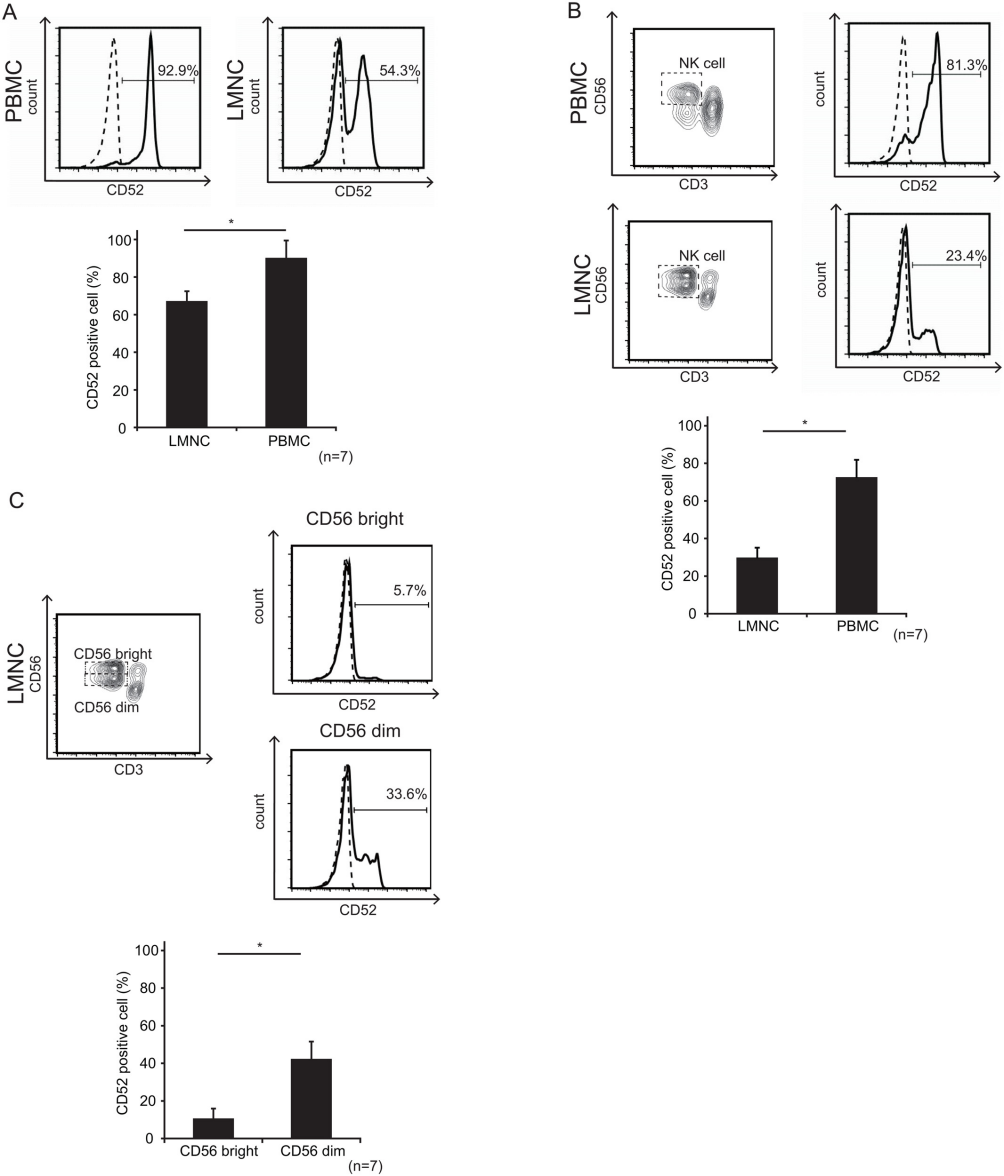
The liver contained a high percentage of CD52^−^ NK cells. CD52^−^ and CD52^+^ NK cells were defined as follows: Cells were gated on the CD3^−^CD56^+^ NK cell, CD3^−^CD56^bright^, or CD3^−^CD56^dim^ populations within singlet and lymphocyte gates. A CD52^−^ gate was set using the isotype control. Data are representative of 7 separate experiments. (A) CD52 expression on LMNCs and PBMCs. CD52 expression levels on LMNCs and PBMCs were evaluated by FCM. LMNCs contain a significantly larger proportion of CD52^−^ cells when compared with PBMCs. The bar graph shows the mean ± SD of CD52^−^ lymphocytes in the liver and peripheral blood (n = 7, *p < 0.05 by Student’s *t*-test) (B) CD52 expression on NK cells in the liver and peripheral blood. CD52 expression on NK cells from the liver was significantly lower than that on NK cells from the peripheral blood. The bar graph shows the mean ± SD of CD52^−^ NK cells derived from LMNCs and PBMCs (n = 7, *p < 0.05 by Student’s *t*-test) (C) CD52 expression on liver CD56^bright^ and liver CD56^dim^ NK cells. CD52 expression levels of each population were calculated by FCM. About 90% of CD56^bright^ liver NK cells (10.2% ± 5.7) did not express CD52. CD52 expression on CD56^dim^ NK cells was significantly higher when compared with that on CD56^bright^ liver NK cells. The bar graph shows the mean ± SD of CD52^−^ cells in each NK cell population (n = 7, *p < 0.05 by Student’s *t*-test).

To analyze whether alemtuzumab affected NK cells, we collected PBMCs and LMNCs by gradient centrifugation from 7 organ donors. LMNCs were isolated from the liver graft perfusate. The proportion of LMNCs extracted from the liver perfusate was almost identical to that of LMNCs collected by enzymatic dissociation, as previously reported [10, 11]. First, CD52 expression of the cells was analyzed by FCM, because alemtuzumab is an antibody that directly binds the CD52 cell-surface antigen. CD52 expression on LMNCs was significantly lower than that on PBMCs (p< 0.05) (Fig 1A). Although almost all liver lymphocytes (T-cells, B-cells, and NKT cells) expressed CD52, only approximately 30% of liver NK cells expressed CD52 (29.0% ± 11.5). This value was significantly lower than that for peripheral blood NK cells (69.9% ± 7.6) (Fig 1B). CD52 expression on peripheral blood NK cells was significantly lower than that of other lymphocytes in peripheral blood (S2 Fig). We also found that most CD56^bright^ liver NK cells did not express CD52 (10.2% ± 5.7). On the other hand, almost half of CD56^dim^ liver NK cells were CD52 positive (41.9% ± 9.7) (Fig 1C).

### Liver NK cells survived even after co-incubation with alemtuzumab

**Fig 2:**
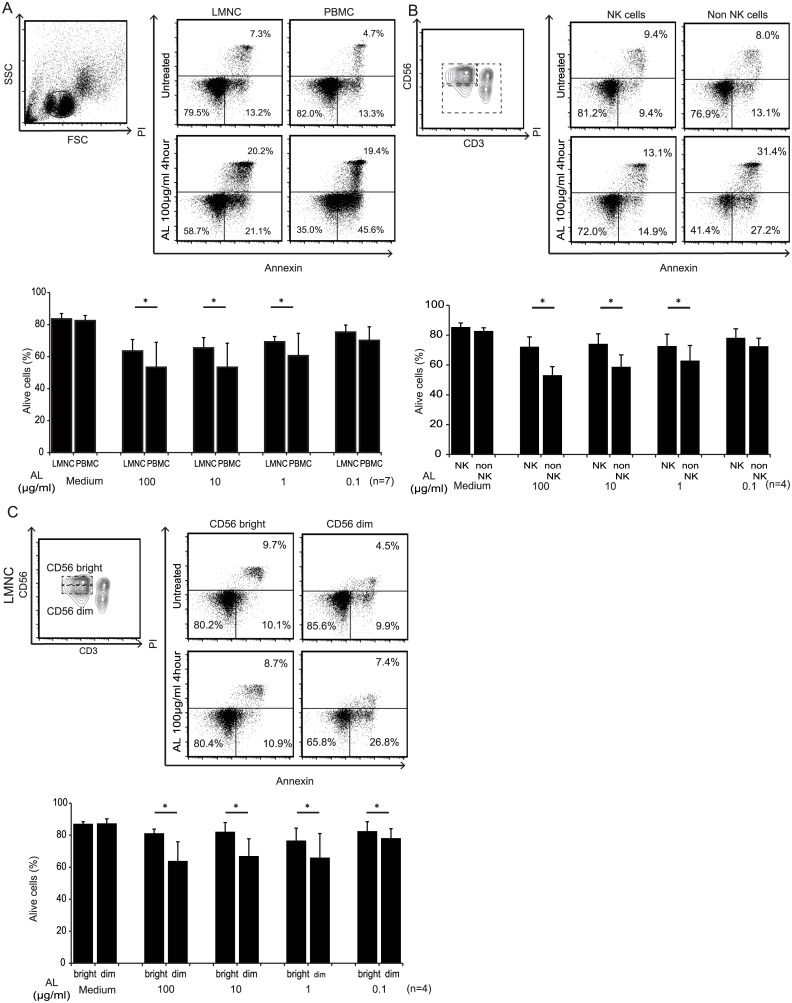
Effect of alemtuzumab concentration on survival of NK cells from the liver and peripheral blood. Cell survival was evaluated using Annexin V and propidium iodide staining. Mononuclear cells were incubated alone or in the presence of alemtuzumab (100, 10, 1, or 0.1 μg/mL) for different time periods (1 and 4 h). The surviving cells were negative for both Annexin V and propidium iodide. (A) The survival rate of LMNCs after co-incubation with alemtuzumab was significantly higher than that of PBMCs for each concentration. The numbers present the proportion of each subset. The dot plots are representative of 7 independent experiments (alemtuzumab; 100 μg/mL, 4-h culture). The bar graphs show the mean survival rate ± SD of LMNCs and PBMCs after 4-h treatment at each concentration (n = 7, *p < 0.05 by Student’s *t*-test). (B) The survival rate of liver NK cells was significantly higher than that of liver-derived non-NK cells (n = 4, *p < 0.05 by Student’s *t*-test). (C) CD56^bright^ NK cells survived after co-incubation with alemtuzumab, compared to CD56^dim^ NK cells (n = 4, *p < 0.05 by Student’s *t*-test).

To analyze the difference in susceptibility to alemtuzumab between PBMCs and LMNCs *in vitro*, the cells were treated with various concentrations of alemtuzumab (0, 0.1, 1, 10, and 100 μg/mL) for different time periods (1 h and 4 h). Co-incubation with alemtuzumab induced cell death in PBMCs in a dose-dependent manner. However, under the same conditions, most LMNCs survived even with co-incubation with alemtuzumab. The survival rate of LMNCs after co-incubation with alemtuzumab was significantly higher than that of PBMCs, at every concentration (Fig 2A). As NK cells comprised a large proportion of LMNCs and most of them were CD52-negative, we wondered whether liver NK cells were less susceptible than liver non-NK cells. As shown in Fig 2B, we observed that the survival rate of liver NK cells was significantly higher than that of liver non-NK cells. Although approximately 30% of liver NK cells still expressed CD52, co-incubation with alemtuzumab did not significantly increase cell death, suggesting no linear relationship between the degree of CD52 expression and cell depletion. Next, we compared CD56^bright^ and CD56^dim^ NK cells because of their significantly different CD52 expression levels. As hypothesized, CD56^bright^ NK cells remained alive, while CD56^dim^ easily began to undergo death or apoptosis (Fig 2C). These results indicated that liver NK cells, especially CD56^bright^ and CD52^−^ NK cells, might survive even after alemtuzumab treatment.

### CD52^+^ and CD52^−^ NK cells from the liver had different FACS profiles

**Fig 3:**
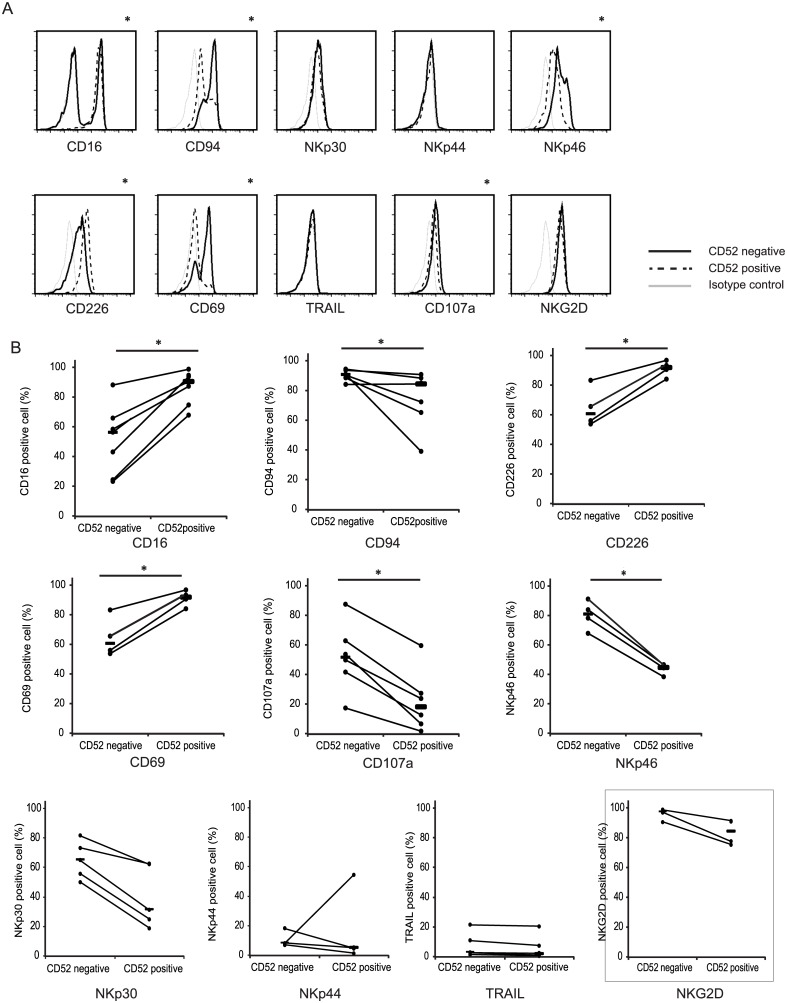
CD52^+^ and CD52^−^ NK cells from the liver had different FACS profiles. (A) Comparison of CD52^+^ NK cells and CD52^−^ NK cells in the liver. The representative histograms of 7 independent experiments are shown for CD52^+^ NK cells (solid line) and CD52^−^ NK cells (dotted line). The gray solid line shows the isotype control. (B) Dot shows the percentage of each surface marker on CD52^-^ and CD52^+^ cells. The solid line indicates mean value in each population and two points connected by dotted line indicate these cells are from same donor. (*p < 0.05 by Student’s paired *t*-test). (n = 4 or 7, *p < 0.05).

Next, we analyzed the FACS profiles of CD52^−^ and CD52^+^ NK cells in the liver. The expression levels of CD69, CD107a, NKp46, and CD94 were significantly higher in liver CD52^−^ NK cells. Liver-derived CD52^−^ NK cells expressed significantly lower amounts of CD16 and CD226. Interestingly, NKp46 expression on CD52^+^ NK cells was significantly lower than that on CD52^−^ NK cells. The expression level of NKp30, NKp44, TRAIL, CD158a, and CD158b were not different between CD52^-^ and CD52^+^ NK cells in the liver (Fig 3A and 3B, and Data not shown). Interestingly, CD69 and CD94 expression levels were significantly higher, and CD16 and CD226 expression were significantly lower in the liver CD52^−^ NK cells when compared with peripheral blood CD52^−^ NK cells. Instead, the phenotypes of CD52^+^ NK cells from the liver and peripheral blood were almost identical (S3 Fig).

### CD52^−^ NK cells exhibited strong cytotoxicity and high IFN-γ production

**Fig 4:**
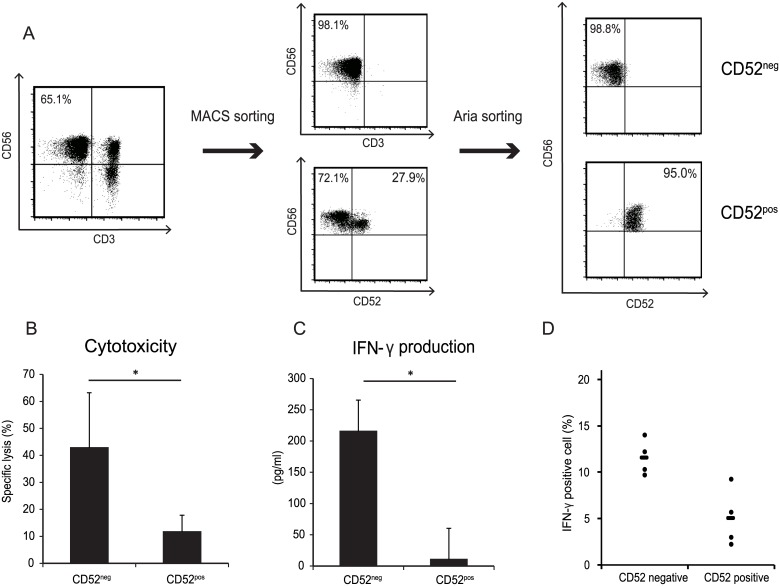
Strong cytotoxicity and IFN-γ production of CD52^−^ NK cells. (A) The CD52 sorting strategy is shown. NK cells were pre-sorted from LMNCs using an MACS NK cell isolation kit and separated into CD52 positive and negative fractions using an FACS-Aria instrument. (B) Cytotoxicity toward K562 target cells was analyzed using a flow cytometry-based cytotoxic assay. The CD52 positive and negative NK cell fractions were stimulated with IL-2 (1000 U/mL) for 48 h. The effector to target ratio was 10:1. Data are presented as the mean ± SD (n = 4, *p < 0.05 by Student’s *t*-test). (C) The IFN-γ level of the culture supernatant in Fig 3B was measured by ELISA. Data are presented as the mean ± SD (n = 4, *p < 0.05 by Student’s *t*-test). (D) The IFN-γ production of both the CD52-positive and -negative NK cell fractions was analyzed by intracellular flow cytometry staining. Freshly isolated LMNCs were treated with leukocyte activation cocktail for 4 h. The numbers indicate the IFN-γ-positive percentage of each population. Data are presented as the mean ± SD (n = 4, *p < 0.05 by Student’s t-test).

Next, we examined CD52^−^ NK cells, which might provide protective immune function after alemtuzumab treatment. To study the function of CD52^−^ NK cells, we tested the cytotoxicity and IFN-γ production of the CD52 fraction sorted from the liver NK cells (Fig 4A). This fraction was cultured with IL-2 for 2 days, and cytotoxicity was analyzed in an FCM-based assay. After 2 days of culture, the CD52, CD56, and CD16 expression levels of both fractions were unchanged. The distributions of CD56^dim^ and CD56^bright^ cells in the two fractions were also not altered (data not shown). When we compared IL-2-activated CD52^+^ NK cells and CD52^−^ NK cells, we found that CD52^−^ NK cells exhibited 4-fold greater cytotoxicity against K562 tumor cells compared with CD52^+^ NK cells (Fig 4B). We analyzed the IFN-γ levels secreted into the supernatant and found that compared with CD52^+^ NK cells, CD52^−^ NK cells cocultured with IL-2 produced 20-fold more IFN-γ (Fig 4C). Using intracellular FCM, we confirmed that CD52^−^ NK cells were the only source of IFN-γ (Fig 4D).

## Discussion

In this study, we showed that CD52 expression on NK cells was lower than that of other lymphocyte groups (S2 Fig). These findings are compatible with those in a report by Rao et al. [14]. Interestingly, we also found that compared to peripheral blood, the human liver contained a larger number of CD52^−^ NK cells, which cannot be targeted by alemtuzumab treatment because of the inability of alemtuzumab to bind. We also showed that liver NK cells exhibited decreased susceptibility to alemtuzumab compared with other lymphocytes *in vitro* (Fig 2B). These findings indicate that liver NK cells might survive even after alemtuzumab-based lymphocyte depletion following organ transplantation. This is important because liver NK cells simultaneously contribute to innate protective functions against microbes while contributing to liver parenchymal cell protection against immune damage.

To the best of our knowledge, this is the first demonstration of CD52^−^ NK cells in the human liver. Interestingly, our results showed that the CD52^−^ NK cells in the liver and peripheral blood have different levels of surface marker expression. CD69 and CD94 expression levels were significantly higher in the liver CD52^−^ NK cells when compared with peripheral blood CD52-NK cells, and liver CD52^−^ NK cells expressed significantly lower amounts of CD16 and CD226. Instead, the phenotypes of CD52^+^ NK cells from the liver and peripheral blood were almost identical (S3 Fig). Every tissue in our body consists of a unique microenvironment that can differentially shape immune reactivity. The liver immune environment is unique because it is continually exposed to gut-derived antigens [15], which may have a role in endowing the liver with some immune privilege [16]. On the other hand, liver NK cells contribute to the protection of liver parenchymal cells against immune damage. The intrinsic “activation” of adaptive and innate cells within the liver may reflect this dual role of ensuring tissue immune privilege and offering antimicrobial protection. The prior exposure of liver innate immune cells to gut-derived microbial products may, in part, explain the altered NK cell phenotypes and behavior in liver NK cells.

Most of the CD56^bright^ liver NK cells did not express CD52 on the cell surface (Fig 1C). Rao et al. observed the same phenomenon for peripheral blood NK cells, a minor population of PBMCs [14]. In a model of transgenic mice expressing human CD52, splenic NK cells expressed little CD52, and lymphocyte depletion was not as profound in lymphoid organs [17]. In humans, CD52 is not expressed on peripheral blood dendritic cells. Similarly, epidermal and small intestinal dendritic cells did not express CD52 and were therefore unlikely to be depleted by alemtuzumab treatment [18]. In our *in vitro* study, LMNCs, which contain abundant CD52^−^ NK cells, appeared relatively resistant to alemtuzumab, whereas circulating PBMCs were depleted in a dose-dependent manner (Fig 2A). Even addition of 20% autologous serum, as a source of complement during the alemtuzumab exposure in vitro did not increase cell death of liver. There is however, good evidence that the major mechanism of alemtuzumab-based lymphocyte depletion is ADCC [19]. Although other interpretations are possible, our results suggest that liver NK cells express little CD52, and are therefore relatively unaffected by alemtuzumab treatment, such that they survive and can carry out their innate protective function.

We also found that liver-derived CD52^−^ NK cells exhibited 4-fold greater cytotoxicity toward K562 tumor cells and produced 20-fold more IFN-γ than liver-derived CD52^+^ NK cells when activated with IL-2 (Fig 4). After 2 days of culture, the CD52 expression levels of both fractions were unchanged. The surface marker expression of IL-2-activated NK cells in both fractions was unchanged when compared with cells prior to activation, except for the expression levels of CD94, NKp30, and NKp44 in CD52^−^ cells, reflecting the fact that NK cells were activated by cytokines. No changes were observed in CD56 and CD16 expression. The distributions of CD56^dim^ and CD56^bright^ cells in the two fractions were not altered after IL-2 activation. These findings indicate that the liver contains high numbers of CD52^−^ NK cells, which are minimally susceptible to alemtuzumab. Liver CD52^-^ NK cells strongly expressed activation marker, NCRs, and NKG2D. NCRs and NKG2D play important roles for regulation of bacterial, parasite and viral infection. The NKG2D system has been reported to provide immunity against infectious disease. NKG2D ligands are not present on the normal cell surface, but are induced in viral infected cells [5]. Some interactions between NCRs and various viruses including the influenza virus were capable of triggering NK cells [6]. Cytokine activation induces expression of IFN-γ and vigorous cytotoxicity in CD52^-^ NK cells (Fig 4B and 4C). Therefore, NCRs and NKG2D expressing CD52^-^ NK cells can prevent microbial infection after cytokine activation, which is likely caused by inflammation or infection.

There are some limitations in our study. First, it is unknown about the possible effect of other immunosuppressive drugs on CD52^−^ NK cells. For organ transplant recipients, alemtuzumab is not the sole immunosuppressant, and additional drugs are required. Second, we assessed the function of CD52^−^ and CD52^+^ NK cells following incubation with IL-2, and showed increased killing and IFN-y production by CD52^−^ NK cells. However, it was unclear whether alemtuzumab can affect the function of CD52^−^ NK cells. We are now conducting experiments to resolve these limitations. Third limitation of this study is that there were no additional data of the patients following liver transplantation. S1 Fig showed that the number of peripheral NK cells do not change during alemtuzumab induction. This raised the question whether peripheral NK cells have a protective role independent from liver resident NK cells or they migrate into the liver during infection.

In summary, we have shown that CD52 expression on NK cells is lower than that of other lymphocytes. In particular, the liver contains a large number of CD52^−^ NK cells. These cells are refractory to co-incubation with alemtuzumab and have robust activity. These findings indicate that CD52^−^ NK cells survive and might protect against infection after alemtuzumab-based lymphocyte depletion.

## Supporting Information

S1 Fig Sequential follow-up of the proportion (A) and the absolute counts/mm^3^ (B) of peripheral blood mononuclear cell subpopulations in liver transplant patients receiving alemtuzumab induction therapy. The subpopulations were assessed by flow cytometry before and after transplant. The open-circle dotted line (CD3^+^ T cells), closed-circle black line (CD3^−^CD56^+^ NK cells), and closed-triangle gray line (CD19^+^ B cells) indicate the mean ± SEM of (A) the proportions and (B) the absolute counts/mm^3^, which were determined by multiplying the respective percentages obtained with flow cytometry by the absolute lymphocyte counts from clinical laboratory reports (n = 17). We gave alemtuzumab, 0.5mg/ kg, one dose, after the liver transplant in the ICU, on Operative day as induction with low dose Tacrolimus. HCV patients were not received this medication.
(EPS)

S2 Fig Expression of CD52 on various lymphocyte populations derived from peripheral blood and liver. The expression of CD52 on NK cells, especially in the liver, was lower than that of other lymphocyte populations. (n = 7, *p < 0.05).
(EPS)

S3 Fig The phenotypes of CD52^+^ NK cells from the liver and peripheral blood were almost identical, instead CD52^-^ NK cells in the liver and peripheral blood have different levels of surface marker expression. The phenotype of CD52^–^ and CD52^+^ NK cell populations derived from Liver and Peripheral blood were evaluated by FCM. (A) The representative histograms of 7 independent experiments are shown for CD52^+^ NK cells (upper) and CD52^-^ NK cells (lower) in peripheral blood (dotted line) and liver (solid line). Gray solid line shows Isotype control. (B) CD69 and CD94 expression levels were significantly higher in the liver CD52^−^ NK cells when compared with CD52^-^ NK cells from peripheral blood. Liver CD52^−^ NK cells expressed significantly lower amounts of CD16 and CD226. Instead, CD52^+^ NK cells in liver and peripheral blood had similar phenotype. Dot shows the percentage of each surface marker on CD52^-^ and CD52^+^ cells. The solid line indicates mean value in each population and two points connected by dotted line indicate these cells are from same donor (n = 4 or 7, *p < 0.05 by Student’s paired t-test).
(EPS)
